# The Role of the Neural Exposome as a Novel Strategy to Identify and Mitigate Health Inequities in Alzheimer’s Disease and Related Dementias

**DOI:** 10.1007/s12035-024-04339-6

**Published:** 2024-07-05

**Authors:** Ravid Granov, Skyler Vedad, Shu-Han Wang, Andrea Durham, Divyash Shah, Giulio Maria Pasinetti

**Affiliations:** 1 Department of Neurology, Icahn School of Medicine at Mount Sinai, New York, NY 10019 USA; 2 Geriatrics Research, Education and Clinical Center, JJ Peters VA Medical Center, Bronx, NY 10468 USA

**Keywords:** Alzheimer’s disease, Artificial intelligence, Machine learning, Neural exposome, Risk factors

## Abstract

With the continuous increase of the elderly population, there is an urgency to understand and develop relevant treatments for Alzheimer’s disease and related dementias (ADRD). In tandem with this, the prevalence of health inequities continues to rise as disadvantaged communities fail to be included in mainstream research. The neural exposome poses as a relevant mechanistic approach and tool for investigating ADRD onset, progression, and pathology as it accounts for several different factors: exogenous, endogenous, and behavioral. Consequently, through the neural exposome, health inequities can be addressed in ADRD research. In this paper, we address how the neural exposome relates to ADRD by contributing to the discourse through defining how the neural exposome can be developed as a tool in accordance with machine learning. Through this, machine learning can allow for developing a greater insight into the application of transferring and making sense of experimental mouse models exposed to health inequities and potentially relate it to humans. The overall goal moving beyond this paper is to define a multitude of potential factors that can increase the risk of ADRD onset and integrate them to create an interdisciplinary approach to the study of ADRD and subsequently translate the findings to clinical research.

## ADRD and the Role of Health Inequities in the Onset and Progression of the Disease

**Fig. 1:**
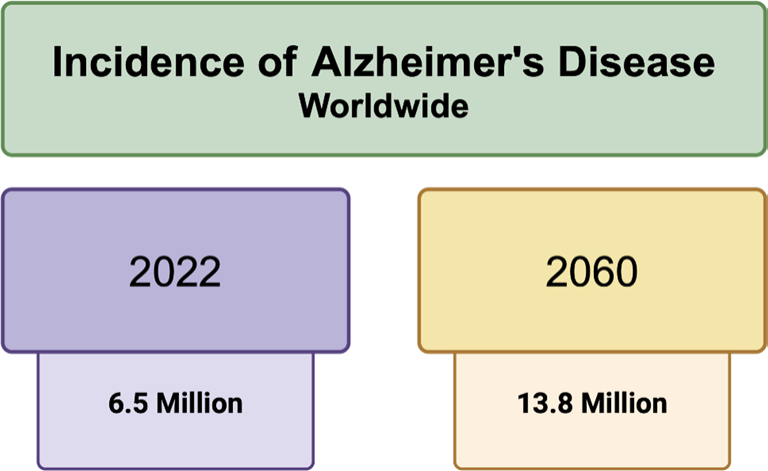
Projected number of people with Alzheimer’s disease from 2022 to 2060 in the worldwide population

Alzheimer’s disease (AD) is a physically progressive illness defined by the decline in memory, cognitive function, and behaviors that worsen over time [1]. Characterized by beta-amyloid plaques, neurofibrillary tangles, and neurodegeneration, AD is ranked as the seventh leading cause of death in the USA and the most common cause of dementia among older adults [1, 2]. Alzheimer’s disease and related dementias (ADRDs) refer to a broader accumulation of the common forms of neurodegenerative illnesses in tandem with cognitive impairment and decline. As the most common cause of dementia, the prevalence recorded in 2022 shows that ADRD affects approximately 6.5 million Americans ages 65 and older, and this number is estimated to rise to 13.8 million by the year 2060 [3] (Fig. 1). Globally, the prevalence of ADRD has spanned to over 55 million people worldwide living with dementia in 2020. These statistics will nearly double every 20 years, ultimately towards affecting 139 million individuals in 2050 [4, 5]. Through this notion, the burden of AD is rapidly expanding with aging populations and increasing numbers, prompting a targeted focus on the modifiable factors that may cause or affect the prevalence, mortality, and morbidity of ADRD. Currently, the etiological mechanisms underlying the neuropathological changes observed in ADRD remain undetermined; however, varying components are at play with regard to influencing ADRD onset and progression.

### Sex Differences in ADRD

An essential component influencing ADRD risk assessment and reduction is sex differences in individuals. There is a critical distinction between sexes regarding ADRD, and understanding the sex-specific brain differences in individuals can allow for a comprehensive understanding of the psychological factors of ADRD. Sex is a potential confounding variable for ADRD incidence with older adults at greater risk for ADRD. A study found that the estimated lifetime risk for Alzheimer’s dementia at age 45 was approximately one in five (20%) for women and one in ten (10%) for men [3]. The risks for both sexes were slightly higher at age 65 [3]. Biologically, brain development, adult brain structure, function, and biochemistry differ by sex [6]. Sex-specific differences in dopaminergic, serotonergic, and gamma-aminobutyric acid (GABA)-ergic markers indicate that male and female brains are neurochemically distinct [6]. Developing deeper insight towards the sex differences in the human brain can provide a foundation for pathophysiological mechanisms and models to guide the production and assessment of sex-specific treatments for ADRD. It is important to distinguish sex from gender, with sex referring to biological differences such as chromosomal, gonadal, or hormonal, and gender referring to psychological and cultural differences between men and women [7]. Both factors are uniquely essential towards the onset and progression of disease [7]. In relation to ADRD, the sex differences in genetics demonstrated from the findings of a large prospective cohort study, published in 2015, confirmed previous case–control reports that women who are positive for the ɛ4 allele of the apolipoprotein E gene (*APOE* ɛ4), the strongest genetic risk factor for ADRD, are at greater risk of developing ADRD than men with this allele [8]. Although sex-differences can elucidate varying genetic components regarding ADRD, it cannot account for the breadth of the other factors underlying ADRD.

### Risk Factors

**Fig. 2:**
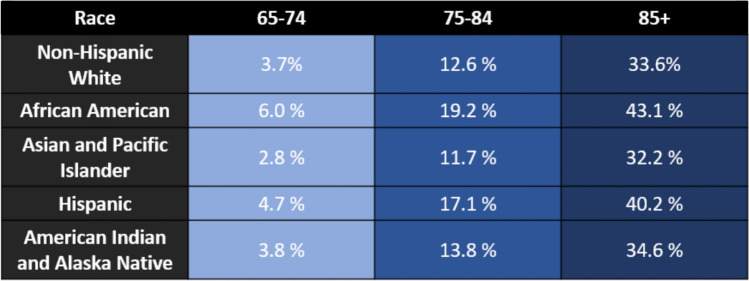
Estimated prevalence of Alzheimer’s disease and related dementias in the US population aged ≥ 65 years, by age, race and ethnicity; United States, 2014. Based on data from Matthews et al., 2019 [12]

There is a suspected broad range of factors affecting individuals’ health, specifically related to the risk of ADRD, and the social determinants of health, including factors such as where one was born, where one lives, their age, and a wider set of conditions that shape an individual’s life [9]. Social determinants of health include factors that are modifiable towards risk assessment and risk reduction of ADRD. To specify, key characteristics comprising social determinants of health affecting ADRD include sex differences, lower socioeconomic status, lower education, manual labor, diet, economic disadvantage, and race and ethnicity [10]. Through this, a study investigated if molecular biomarkers of ADRD can be varied by race and found significant differences in the cerebrospinal fluid (CSF) concentrations of tau protein between African American and White individuals [11]. The implications of this study suggest that racial differences in ADRD biomarkers could have possible race-dependent biological mechanisms that contribute to the expression of the disease [11] (Fig. 2). There were significant differences in *APOE* ε*4* for both CSF total tau and phosphorylated tau depending on race, ultimately suggesting that ADRD risk variants could have differing expressions depending on race [11]. The relevancy of this study demonstrates how race must be included as a heightened risk factor in association with ADRD, and that molecular biomarkers of ADRD should adjust for race [11]. In a broader notion, high levels of stress are linked to a greater probability of developing ADRD. Stress including racial discrimination leads to a higher risk that can also impact other social determinants such as education, employment, and residency [11]. Such risk factors can impact the health inequities in individuals, potentially leading to psychosocial detriments that can affect the onset and progression of neurological degeneration.

### Psychological and Social Implications of Health Inequities in ADRD

The scope of the factors at play regarding ADRD extend to the health inequities affecting individuals psychologically and socially. Defined by the World Health Organization (WHO), health inequities are the systematic differences in the health status and outcomes of different population groups [13]. Such inequities have significant social and economic costs both to individuals and societies. These differences in health statuses and resources between varying populations cause instability in available resources. Differing health statuses arise from the social conditions in which people are born, grow, live, work, and age [13]. The importance and reasoning for studying health inequities is to explain the variation in etiology and onset of ADRD in individuals from varying populations to make sense of interventions that can be developed in response. Specifically, regarding disadvantaged communities, residency in disadvantaged neighborhoods has associations with adverse health exposures and outcomes, and may increase risk for cognitive impairment and dementia [14]. ADRD disproportionately affects historically underrepresented and socially disadvantaged populations [15]. Living in socioeconomically disadvantaged neighborhoods has been shown to negatively affect health and increase the risk of pathologies such as higher rates of cardiovascular diseases, diabetes, ADRD, and premature mortality [15]. The psychological implications of health inequities are associated with an increase in stress levels with regards to access to food, safety, and education. Defined as the Neural Exposome, the social vulnerability as a result of social inequities are affected by environmental conditions [16]. The significance of studying health inequities in relation to the psychological and social implications on ADRD is how environmental, behavioral, and internal factors compile together to increase the risk of developing ADRD. The social stress of food insecurity manifests as physical stress neurologically affecting the individual that builds upon other factors from living in disadvantaged communities. Psychological and social components work in tandem with one another and it would not be of benefit to researchers to isolate one from the other. These factors compile into what is defined as the Neural Exposome, affecting ADRD risk and suggesting that disadvantaged social exposure could be associated with late-life cognitive impairment [17].

### The Association Between the Neural Exposome and ADRD

**Fig. 3:**
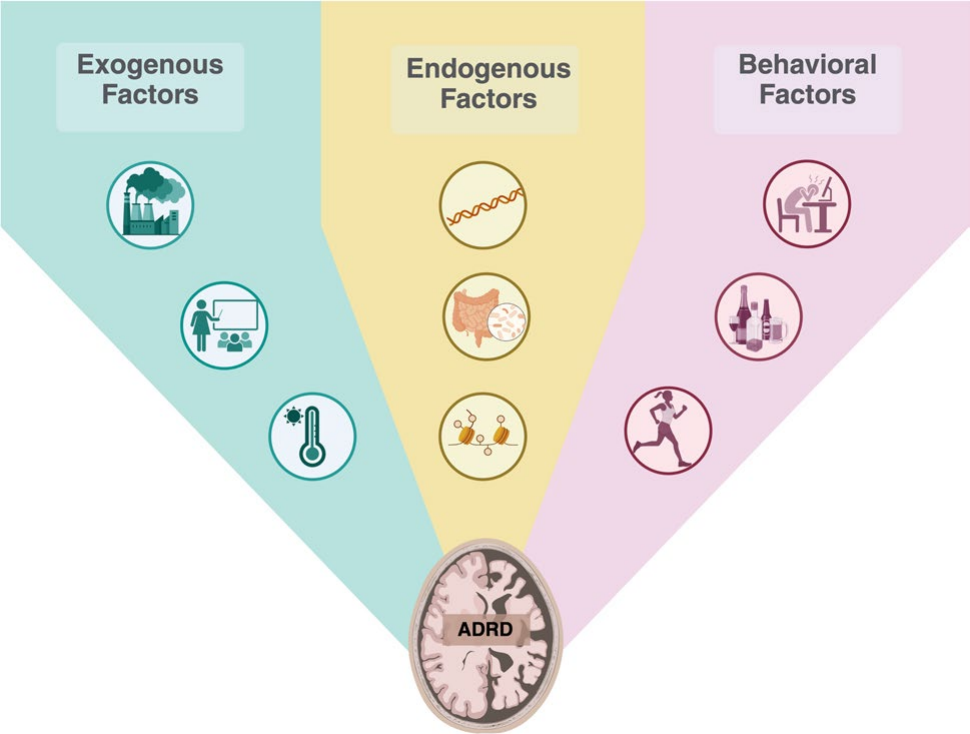
Infographic depicting the neural exposome factors: exogenous, endogenous, and behavioral. These factors have an impact on ADRD as seen by the infographic in which the three factors lead into the ADRD brain image. Exogenous factors include climate change factors, education, and factors that affect disadvantaged communities like socioeconomic status, housing location, neighborhood resources, etc. Endogenous factors include genes/epigenetics, microbiome, metabolism, and pre-existing conditions. Behavioral factors include psychological factors including stress, lifestyle, and drugs/alcohol

**Fig. 4:**
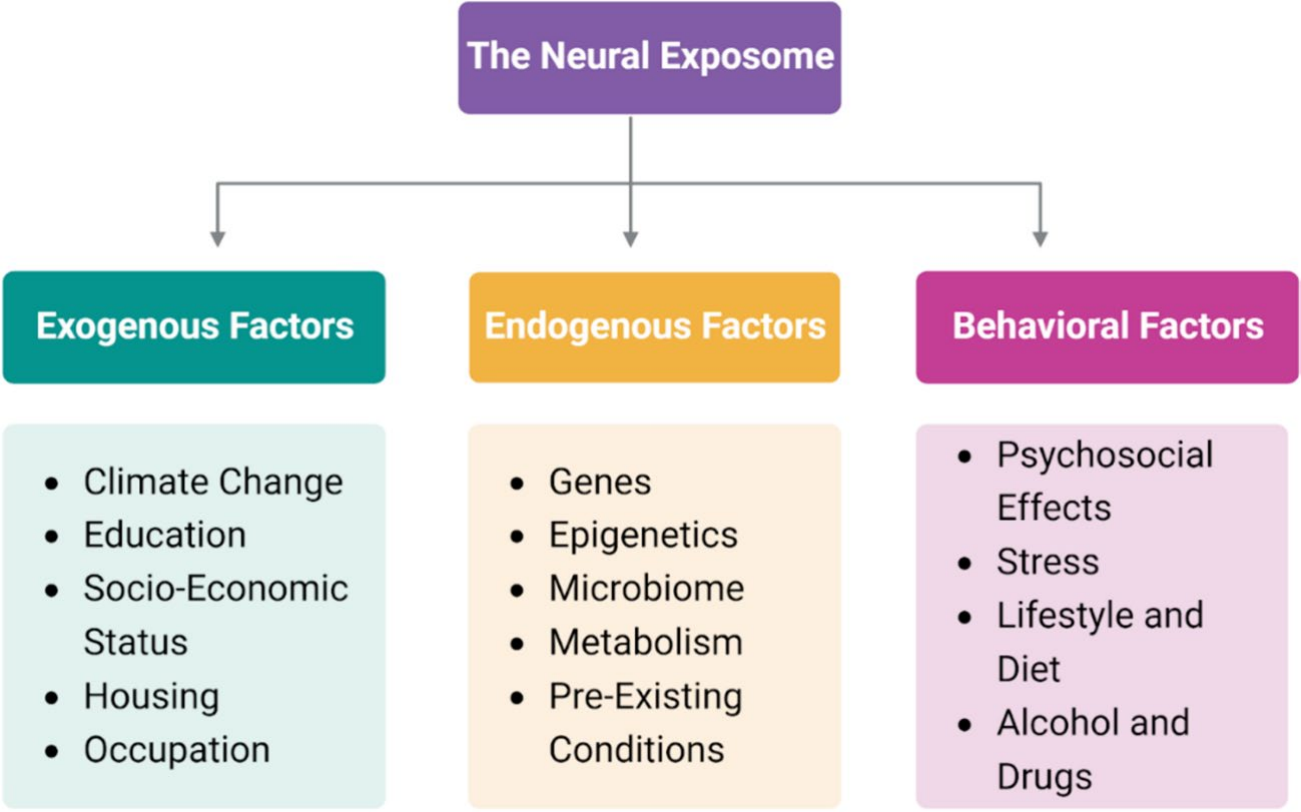
The neural exposome is made up of exogenous, endogenous and behavioral factors. Exogenous factors include climate change, education, socio-economic status, housing, and occupation. Endogenous factors are genes, epigenetics, microbiome, metabolism and pre-existing conditions. Behavioral factors incorporate psychosocial effects, stress, lifestyle and diet, and alcohol and drugs. Together all these factors make up the neural exposome and can be used to study pathology in neurodegenerative diseases such as Alzheimer’s disease and related dementia

As discussed, ADRD cannot be attributed to genetic factors alone; rather, accounting for the neural exposome substantiates a broader depth of exploring the risk factors related to ADRD, and poses as a quantitative framework for experimentally approaching risk assessment in ADRD. The Office of Neural Exposome and Toxicology Research (ONETOX) led the initiative of advancing knowledge of internal and external exposures that affect brain and nervous system health over the lifespan of an individual’s life, defined as the neural exposome [18]. Specifically, the neural exposomes are the exposures that impact and influence neurological disease and disorders, including three factors: exogenous (environmental and chemical), endogenous (biological), and behavioral (psychosocial) spanning from in utero to later in life [18]. The goals of ONETOX include enhancing research regarding the neural exposome on nervous system diseases and disorders by providing information and tools to integrate various scientific disciplines for collaboration. This is driven by the incentive to understand how exposomic factors affect brain and nervous system health [18]. The neural exposome represents the summation of exposures in an individual’s life since conception, allowing for the investigation and identification of risk factors that can predispose and predict ADRD (Fig. 3). Knowledge of the exposomic risk factors and their clinical relevance is the key to unlocking a more holistic approach to disease prevention and more effective and personalized interventions (Fig. 4). In this paper, we will discuss the neural exposome factors and each factor’s relationship to ADRD.

## Exogenous Factors

### Climate Change

One of the emerging fields relating exogenous factors with ADRD has been environmental changes, including climate change. To investigate this field closely, climate change can be sectioned into “climate-sensitive” exposures which include adverse conditions and acute hazards which when taken together affect the environments in which individuals live [19]. In this paper, we will focus on “climate-sensitive” exposures such as extreme heat and air pollution. According to the WHO, climate change will presumably cause an additional 250,000 deaths each year between the years 2030 and 2050, and of these deaths, 38,000 will be as a result of elevated heat exposure in the elderly [20]. Additionally, those suffering from neurodegenerative disorders, specifically dementia, are at higher risk from exposure to heat waves [21]. Specifically, this link has shown higher hospital admission rates in areas with higher temperature, thus showing patients with dementia are more affected by rising temperatures [22]. Furthermore, a nationwide study conducted in China showed that the fluctuations in temperature, extreme heat and extreme cold, are associated with neurodegenerative diseases proposing a link that those experiencing the brunt of the climate change impact may be more likely to develop these disorders [23]. In addition to ADRD, increased heat may cause several adverse health conditions which may affect and even exacerbate the onset and progression of ADRD [24, 25].

### Air Pollution

Another risk factor linked with climate change is air pollution, which can diminish life expectancy by about 2 years according to the Health Effects Institute in Boston Massachusetts [26]. A recent systematic review found that exposure to some forms of air pollution, PM2.5, was significantly and positively associated with dementia while other forms may need more investigation [27]. Similarly, there has been substantial evidence linking proximity to areas with greater air pollution and increased cognitive decline [28, 29]. There are several types of air pollution, for example, particulate matter (PM), which can be found in different concentrations [30]. High PM exposure has been linked to adverse abilities on cognitive tests in the elderly [31]. While it has been repeatedly found that PM affects cognitive abilities in elderly, the results in adults and children have shown varied results. A set of studies examining adults (average age of 37) shows PM did not impair cognitive abilities [32, 33]. On the other hand, a novel study found a strong correlation between accumulation of amyloid beta and traffic-related pollutants in the 45–75 age adult cohort [34]. A cross-sectional study investigating traffic-related air pollutants in children showed a positive correlation between pollutant exposure and behavioral changes [35]. These results potentially suggest that vulnerable populations may be more greatly influenced by exposure to air pollution but data on young and middle-age adults is still limited and needs to be investigated further. Other forms of air pollution that have been linked with ADRD include SO_2_ and NO_2_ [36, 37]. However, a lack of consensus exists in the literature regarding which gasses lead to ADRD, and to date, studies have been unable to confidently establish a causal effect. As one review established, a meta-analysis becomes difficult due to a variety of study methodology and investigation criteria for air pollution and that should be noted when considering results associating air pollutants with ADRD diagnoses [38]. In accordance with this, the neural exposome can greatly affect ADRD progression and onset through exogenous risk factors such as climate change.

### Education

Previous studies have indicated that lack of education is a risk factor for ADRD pathology [39, 40] even when adjusting for the number of *APOE* ε4 alleles [41]. The “reserves theory” serves as an explanation for why education presents as a risk factor for ADRD, and suggests that the more educational experiences one participates in, the more cognitive and neurological “reserves” one has. These “reserves” allow for the brain to compensate for ADRD pathology in the early periods of the disease [42]. This can further be explained through animal models, a topic that will be elaborated on later in this article, yet a study has shown the correlation between education and increased synaptic connections which can serve as “reserves” [43]. Some studies suggest that the more education one has, the better equipped one is to take cognitive tests but a consensus is missing as others have noted that less education is correlated with bad test-taking strategies creating a potential investigation bias [44]. Nevertheless, literacy may be an alternative exogenous neural exposome risk factor, especially for minority groups [45]. Several studies found that literacy is a preventative factor for ADRD, suggesting that illiteracy influences cognitive decline [46, 47]. A deficiency in synapses because of a lack of stimulation caused by illiteracy could account for a decrease in “reserves” which subsequently supports the “reserves theory” [48]. Consequently, education and more specifically literacy serves as a relevant risk factor as an exogenous neural exposome trait in the onset and progression of ADRD.

### Disadvantaged Communities

Disadvantaged communities face several exogenous neural exposome risk factors including socioeconomic status (SES), housing and neighborhood resources, employment, and food insecurity, all of which have been shown to be risk factors of ADRD [49]. Several studies have shown that low SES and wealth are correlated with increased incidence of ADRD and accelerated cognitive decline while high SES has been shown to decelerate the time of diagnosis [49–51]. Economic hardship is correlated with increased cognitive decline [52]. Interestingly, a longitudinal study found that high SES in childhood is associated with better memory at baseline and slower cognitive decline throughout the lifetime [53]. In addition to SES, disadvantaged communities are influenced by their housing and neighborhood resources. It has been shown that economically disadvantaged neighborhoods are associated with increased cognitive decline [54, 55]. Furthermore, a study which focused on the resources supplied by the neighborhood, such as community centers and proximity to public transport, found that neighborhoods with lower resources had populations with increased cognitive decline [56]. As aforesaid, climate change exposure is a major risk factor for ADRD onset and progression, and this risk is even greater for disadvantaged communities which are more likely to be found near highways and industrial factories and thus have a greater risk for exposure [57]. Thus, housing and neighborhood resources are risk factors for ADRD and cognitive decline. Employment is an additional exogenous neural exposome factor that poses a risk for ADRD. A study performed on a US cohort found that continued lifetime occupation decreased the risk for ADRD [58]. However, a study performed on a different cohort found that this risk was dependent on education level [59]. Additionally, several studies have concluded that manual occupations, in comparison to intellectual occupations, are correlated with an increased risk in cognitive decline [60, 61]. Therefore, the type of occupation and the continued practice of that occupation may be an invaluable neural exposome factor for ADRD. Food insecurity is defined as “whenever the availability of nutritionally adequate and safe foods or the ability to acquire acceptable foods in socially acceptable ways is limited or uncertain” [62]. Early life food insecurity has been shown to double the chances of dementia in later life [63]. In addition, increased cognitive decline was shown to be influenced by food insecurity in a Boston-based longitudinal study [64]. Furthermore, the cognitive decline measured was more pronounced for those living in poverty. However, this decline could also be explained by poor diets which are more pronounced in disadvantaged communities [65], demonstrating the urgency and need for expanding research in this field. Disadvantaged communities face increased exposure to exogenous neural exposome factors increasing their risk of onset and progression of ADRD, especially in minority groups. Consequently, research models need to accordingly model and account for the differences these populations experience.

## Endogenous Factors

### Genetics/Epigenetics

The neural exposome includes endogenous or internal factors that influence ADRD. In accordance with the ONETOX definition of the neural exposome, genes and epigenetics are considered to be endogenous factors [18]. One type of ADRD, early-onset Alzheimer’s disease (EOAD), afflicts about 5% of the population diagnosed with ADRD, while mostly heritable, the known genes associated with this prognosis are amyloid precursor protein (APP), presenilin 1 (PSEN1), and presenilin 2 (PSEN2) [66]. These genes show almost 100% penetrance and are autosomal dominantly inherited [67]. Most ADRD cases are generally caused by environmental and genetic factors combined, as each alone is not enough to explain the etiology [67]. One major risk factor for sporadic late-onset Alzheimer’s disease (LOAD) is the APOE gene, and specifically the ε4 allele, because of its location on chromosome 19 in a peak linkage region [68]. However, less than 50% of sporadic LOAD cases carry the APOE ε4 allele, thus suggesting that other genes are involved [69]. Several other gene mutations have been found to be present among populations with LOAD; however, these genes alone are not thought to be the cause for the disease, but rather the interaction with the environment, biology, and epigenetics that cause the onset and progression of ADRD [66, 67]. Thus, furthering the need to create models that include both exogenous and endogenous neural exposome factors. Epigenetics, an endogenous neural exposome factor, has also been linked to ADRD pathology. For example, an increase in DNA methylation in several regions of the brain has been measured in ADRD patients, including the hippocampus, the temporal cortex, and the temporal gyrus [70–72], while other studies found general decreases in DNA methylation in ADRD patients in the prefrontal cortex and locus coeruleus [73, 74]. It has been found in the post-mortem brains, CSF, and blood monocytes of ADRD patients that miRNA are expressed differently [75]. This is of importance as many of these miRNAs have been linked to coordination of amyloid production and clearance [76]. In addition, several histone modifications have been linked to ADRD pathology of which these modifications have been associated with neuroplasticity genes, which influence learning and memory [77]. Consequently, two highly researched risk factors of ADRD are genetic and epigenetic alterations; however, these are not the only endogenous neural exposome factors that need to be taken into consideration in the study of ADRD onset and progression.

### Microbiome

The microbiome, specifically its dysbiosis, is an important neural exposomic factor to discuss as a risk factor for ADRD. A study found that ADRD patient populations have a decreased amount of *Firmicutes* and *Actinobacteria*, and an increase in *Bacteroidetes* in comparison to control groups [78]. In addition, these changes have also been shown in transgenic ADRD mice [79]. These studies are pertinent to ADRD since the major outer membrane of *Bacteroidetes*, which is a gram-negative cell, contains lipopolysaccharide (LPS) [80]. LPS can cause systemic inflammation and promote the release of pro-inflammatory cytokines [80]. The release of pro-inflammatory cytokines can cause neuroinflammation which in turn can cause increased permeability of the blood–brain barrier (BBB) and increased cell infiltration to the brain; eventually, this can cause neurodegeneration [81]. LPS has been correlated with ADRD pathology. A study found that co-incubating LPS with amyloid β (Aβ) peptide led to increased amyloid fibrillogenesis [82]. Another study found that continual injection of LPS in mice caused an increase in amyloid deposition and tau-related pathology [83]. Furthermore, it has been suggested that a decrease in the protection of the intestinal wall may cause a “leak” of LPS into the brain further exacerbating the ADRD pathology and this has been shown in a study which found elevated fecal calprotectin levels in patients diagnosed with ADRD, thus suggesting a leaky gut [84]. This could lead to increased exiting of LPS and other harmful substances from the gut to the circulatory system and eventually to the brain furthering the onset and/or progression of ADRD [85]. Interestingly, a study found that transgenic ADRD mice kept in a germ-free environment showed lowered cerebral amyloid deposition when compared with the conventionally kept mice furthering the notion that the microbiome may affect AD pathology [79]. Thus, the microbiome and more specifically the increase of *Bacteroidetes* in the microbiome of ADRD patients is an important endogenous neural exposome factor.

### Metabolism

Another endogenous neural exposome factor is metabolism, specifically energy metabolism. The brain consumes about 20% of body energy, while it is only 2% of the total body weight [86], thus making the brain very susceptible to changes in energy metabolism. Dysregulation of energy pathways has been linked to ADRD as it causes synaptic failure and cognitive impairments [87]. Neuroimaging studies have shown that the brain regions that are the most susceptible to Aβ and tau aggregation are the brain regions which have increased glucose consumption from an early age and are especially reliant on glucose for normal brain function [88–90]. In addition, brain glucose hypometabolism has been repetitively proven to be a symptom of ADRD even during the preclinical stage [88]. One major metabolism factor affected in ADRD is the mitochondria, the organelle responsible for energy production, which has been shown to have altered morphology, decreased function in enzymes associated with the tricarboxylic acid cycle, and diminished cytochrome c oxidase (COX) activity [91–95]. Studies have shown that Aβ accumulates within the mitochondria, interacting with a mitochondrial protein named Aβ-binding-alcohol-dehydrogenase (ABAD), and thus, causing a decrease in COX activity and an increase in oxidative stress [96–98]. An additional metabolism factor that is altered in ADRD patients is brain insulin and brain insulin growth factor (IGF) of which have shown to cause an increase in the activity of kinases that phosphorylate tau, increased expression of APP and accumulation of APP-A, increased levels of oxidative, the generation of reactive oxygen and reactive nitrogen species, mitochondrial dysfunction, and activation of pro-inflammatory and pro-death cascades [99]. Consequently, glucose hypometabolism, mitochondrial dysfunction, and IGF dysfunction are some of the major endogenous neural exposome factors, and further research should focus on the link between these changes and other neural exposome factors.

### Pre-existing Conditions

Pre-existing conditions, such as cardiovascular diseases, obesity, and type-2 diabetes, are additional endogenous neural exposome risk factors for ADRD. In general, the brain receives about 20% of the body’s total oxygen making the brain particularly vulnerable to loss of oxygen, or otherwise known as impairment of cerebral perfusion, which can occur in cases of heart failure [100]. Cerebral hypoperfusion can cause a metabolic energy crisis in the brain leading to acidosis and oxidative stress [101]. An acidic environment can lead to hyperphosphorylation of tau proteins, through the activation of lysosomal enzymes, and these hyperphosphorylated tau proteins can cluster and cause neurofibrillary tangles [102]. Thus, lack of oxygen to the brain due to heart disease can implicate a series of events that can lead to increased ADRD symptoms. Additionally, hypertension exacerbates the risk for ADRD as it causes cerebral microinfarcts, lacunar infarcts, macro-bleedings, and micro-bleedings, and has been associated with white matter alterations and Aβ accumulations which all pose as risk factors for ADRD [103–105]. In addition to hypertension, obesity has also been linked to increased cognitive decline and poses a risk factor for ADRD [106, 107]. Furthermore, the combination of obesity and hypertension has shown to potentiate a decrease in cognitive abilities in several different cognitive tests in humans [108]. An additional pre-existing endogenous neural exposome risk factor for ADRD is type-2 diabetes (T2D) as it almost doubles the risk for dementia and ADRD [109]. As aforementioned, it has been shown that insulin signaling and resistance are linked to ADRD, and T2D is characterized by the body’s resistance to insulin [110]. Furthermore, studies have shown that diets which induce T2D in animal models of ADRD cause formation of Aβ plaques [111–113]. Finally, hyperglycemia, noted as elevated blood glucose levels as occurs in T2D, has been associated with increased risk of ADRD, faster development of ADRD from mild cognitive impairment (MCI), and an increased aggregation of Aβ [114–116]. Consequently, pre-existing conditions affect the progression and onset of ADRD and are an important endogenous neural exposome factor for future research.

## Behavioral Factors

### Psychological Stressors

The first behavioral factor of the neural exposome we will discuss is psychological stressors that include stress, mental health, and social isolation and how they influence ADRD onset and progression. Several studies have linked stress and the pathology of ADRD in animal models [117–119]. Stress has been shown to increase the expression of APP, accumulation of Aβ, and increase the phosphorylation of tau proteins leading to exacerbation of neurofibrillary tangles production [120]. This can be translated in humans to mental health concerns and increased stress in life. Animal models such as chronic unpredictable stress (CUS) have been proven to cause depressive-like behaviors in mice which can be translated to human forms of depression [121]. In terms of mental health, in humans, it has been found that those who experience late-life depression have a higher risk for developing ADRD [122]. Furthermore, studies have found that early life trauma is associated with almost double the risk for ADRD in later life, and evenmore, PTSD in veterans has also been shown to increase this risk [123–126]. Another risk factor that influences ADRD pathology is social isolation which has been shown to influence mental and physical health as well [127]. It has been shown in both humans and in animal models that social isolation has been linked to worsened memory and increased plaque formation, and in humans increased cognitive decline [128–131]. Accordingly, stress, mental health, and social isolation all may play a role in ADRD pathology.

### Lifestyle

To develop a comprehensive understanding of the behavioral factors of the neural exposome, lifestyle choices play an essential role in the risk assessment and risk reduction regarding ADRD [132]. Understanding individuals’ lifestyle choices, including exercise, diet, and nutrition, allows for more exhaustive means of predicting and preventing ADRD [132, 133]. The most prevalent factor made, voluntarily or involuntarily, under the category of an individual’s lifestyle is through their dietary choices. Consequently, there could be potential implications of specific dietary conditions being associated with increased ADRD risk. From a study conducted by the Chicago Health and Aging Project, an association between the factor of saturated fat intake and risk of developing ADRD was investigated; the findings concluded that individuals with increased saturated fat intake had twice the risk of developing ADRD [132]. To that effect, similar studies have examined and found that high saturated fat intake increases the rate of decline in cognitive abilities with age [132]. Through this understanding, regarding the neural exposome as a tool for developing strategies for ADRD risk assessment is essential, because a multitude of factors dictate an individual’s health. Considering how saturated fat intake increases the risk of cardiovascular disease and T2D, these two diseases in turn can increase the risk of ADRD [132]. Mechanistically, ingesting saturated fats can increase ADRD risk because the APOE ε4 allele produces a protein that plays a key role in cholesterol transport. This increase in blood-cholesterol concentrations could then potentially cause Aβ aggregation in the brain [132]. To a similar extent, the role of exercise is an important factor for examining its relation to ADRD. Previous studies have found that exercise can act as a preventative measure for reducing the risk for developing ADRD by improving brain blood flow, increasing hippocampal volume, and improving neurogenesis [133]. Likewise, exercise for preventive means can promote cognitive function and reduce cortical decay in the elderly [133]. A German population study was conducted to demonstrate, over a 14-year timeline, how regular physical activity could reduce the risk of developing ADRD, and the results revealed that the individuals had a greater performance on neuropsychological tests [133]. Regular exercise over a lifetime is notably more significant towards reducing ADRD risk in individuals because it enhances the endurance of cells, tissues, and organs to oxidative stress, energy metabolism, and vascularization, all of which constitute important inducers of neurogenesis, muscle development, memory improvement, and brain plasticity [134]. An additional lifestyle risk factor of interest in ADRD pathology is the use of drugs and alcohol. Benzodiazepines (BZ) facilitate the inhibitory activity of the neurotransmitter GABA on its receptor and therefore are used to treat in small doses insomnia and in higher doses anxiety [135, 136]. Conflicting research regarding the use of BZ in elderly patients have been reported as they pose both beneficial and unbeneficial effects [136–138]. While insomnia and anxiety can have adverse effects such as stress which have been linked to ADRD pathology as aforementioned, there are other viable treatments for these cases that can be used until further research establishes the link of ADRD and BZ. In addition to BZ, alcohol poses a potential risk for ADRD as well. Long-term alcohol abuse can lead to various health issues, such as high blood pressure, liver cancer, and also ADRD [139–142]. A study containing a cohort of patients from New York, Boston, Baltimore, and Paris with early AD that were followed for up to about 19 years found a trend between the cognitive assessment and the patients’ alcohol intake [142]. It was suggested that heavy drinkers had a significantly faster cognitive decline [142]. Importantly, one factor that needs to be taken into account is that lifestyle factors are variable depending on the individuals’ biology. Nonetheless, the neural exposome should be considered in the investigation of ADRD pathology.

## The Neural Exposome and the Relationship Between Several Factors

While we have demonstrated how each factor can play a role in ADRD as different risk factors, the cooperation between different neural exposome factors must be studied in future research in order to extensively understand the disease and its onset and progression. To exhaustively research the factors associated with increased risk of ADRD, it would be ineffective to individually study the endogenous, exogenous, and behavioral factors; rather, the goal of the neural exposome is to integrate these factors to understand individuals’ lives and experiences. Humans, unlike mice, cannot be separated and tested in specific environments which highlights the importance of understanding how the overall effects of the neural exposome as a whole affects ADRD onset and progression. Additionally, research must take into account the differences between different populations, especially disadvantaged communities and minorities which have been shown to be at a greater risk for ADRD. A recent study on an Australian cohort found a correlation between low socioeconomic status, unhealthy diets (diverging from the Mediterranean diet), and obesity [143]. This study provides beneficial insight towards several neural exposome factors and finding a relationship between the factors, while they did not check for ADRD progression, all these factors together pose as risk factors for ADRD and so open a field for future research to focus on. Another paper found that T2D onset has been associated with climate change factors like increased temperature and that both T2D and climate change are affected by behavioral and exogenous neural exposome factors such as diet, exercise, and urbanization [144]. This further emphasizes the urgency towards understanding how several factors play a role in disease progression and onset and finding new pathways for future research.

## Applications of the Neural Exposome in ADRD Using Animal Models

Evidently, non-genetic factors play a role in the development of ADRD, and as such there is a need to develop animal models to further explore the role of the neural exposome in ADRD onset and progression. Although epidemiological data and human cohort studies have identified associations between environmental exposure and disease initiation and progression, there is a gap in knowledge relating to the mechanistic underpinnings that explain these observations. The neural exposome is multi-faceted, as it accounts for endogenous, exogenous, and behavioral risk factors, and its study as it is related to ADRD requires novel approaches and study designs to recapitulate ADRD. The neural exposome can be applied to animal models in order to test several different exposomic factors to gain insights into the effects of the factors on ADRD, as well as to model disadvantaged communities. Past studies using traditional ADRD mouse models, heavily reflecting Aβ and tau pathology, have already discovered links between exposomic factors such as educational enrichment, particulate matter and diesel engine exhaust exposure, gut microbiome health, exercise, and diet and their effects on ADRD aggregation and symptoms [145–147]. The study designs required unprecedented approaches in order to model exposomic factors.

### Education and Enrichment

Based on past epidemiological studies, there has been an emerging association between higher levels of education and lower risk of developing dementia [148]. Studies have attempted to recapitulate these observations in transgenic ADRD mice to further elucidate these connections, and ultimately extrapolate the findings to how adult education could help inhibit dementia [148, 149]. In one study, an enriched environment was simulated by placing ADRD mice in a cage that included two running wheels, plastic play tubes, cardboard boxes, and nesting materials that were rearranged in order to provide a source of stimulation, and allocating three times the amount of space for each mouse compared to that of control mice kept in colony cages [148]. Ultimately, it was found that exposure to an enriched environment enhanced cognitive performance in all genotypes tested, both transgenic ADRD mice and non-transgenic mice. In the presence of beta-amyloid accumulation, ADRD transgenic mice exhibited attenuation of cognitive deficits [148]. Strikingly, although environmental enrichment induced amyloid pathology, cognitive function had improved under these conditions [148]. Similar findings were found in another study where an educationally enriched environment was simulated by placing ADRD mice in a housing environment with passageways, lofts, running wheels, toys, and novel habitats [150]. Environmentally enriched mice were shown to have superior overall cognitive performance when compared to control mice [150].

### Airborne Pollutants and Particulate Matter

To study climate changes in ADRD, studies have focused on developing experiments to test various air pollutants on ADRD mice. For instance, one study exposed an ADRD transgenic mouse model to World Trade Center Particulate Matter (WTCPM) collected from the September 11th, 2001 attacks. The results found that mice exposed to WTCPM had impairments in spatial and recognition memory, both short term and long term [151], suggesting that airborne pollutants can exacerbate memory impairment and accelerate cognitive impairment. Other studies have focused on the role of diesel engine exhaust (DEE) exposure on ADRD by exposing ADRD transgenic mice to DEE. DEE exposure not only impaired motor function in the transgenic mice, but also led to an observed acceleration Aβ plaque formation [152]. The results of the study suggest that long-term exposure to air pollutants can promote ADRD development. PM is another air pollutant of interest in the study of ADRD as it was found that transgenic ADRD mice exposed to nanoparticulate matter (nPM) had increased levels of cerebral Aβ and Aβ deposits that were exacerbated through APOE ε4 [153]. And so, climate change, a neural exposome factor, in different forms, has been shown to possibly exacerbate ADRD onset and progression in mice models; however, the relationship between this exogenous factor and other factors has not been well established.

### Microbiome, Exercise, and Diet

Various experimental approaches have been developed to study the evident role of the gut microbiome on ADRD onset and progression. One study in particular linked exercise and probiotic supplementation to the attenuation of ADRD in mice, through mechanisms involving the microbiome by administering a combination of exercise training and probiotic treatment to an ADRD transgenic mouse model [154]. The study found that spatial memory improved in the mice that received the exercise and probiotic treatment [154]. Fecal matter transplantations (FMTs) remain a valuable experimental approach to study the role of the gut microbiome in ADRD. One study transplanted the gut microbiota derived from an ADRD transgenic mouse model to a control mouse which led to cognitive impairment, through decreased neurogenesis and increased neuroinflammation in the hippocampus of the control mice, ultimately resulting in memory impairment [155]. The results of another study demonstrated how fecal microbiota from WT mice led to the amelioration of AD pathology when transplanted into a transgenic mouse model [156]. Ultimately, these studies underscore the importance of studying neural exposome factors in relation to ADRD, as they may explain underlying pathological features of ADRD.

### Development of LOAD Mouse Models for Future Studies of the Neural Exposome

**Fig. 5:**
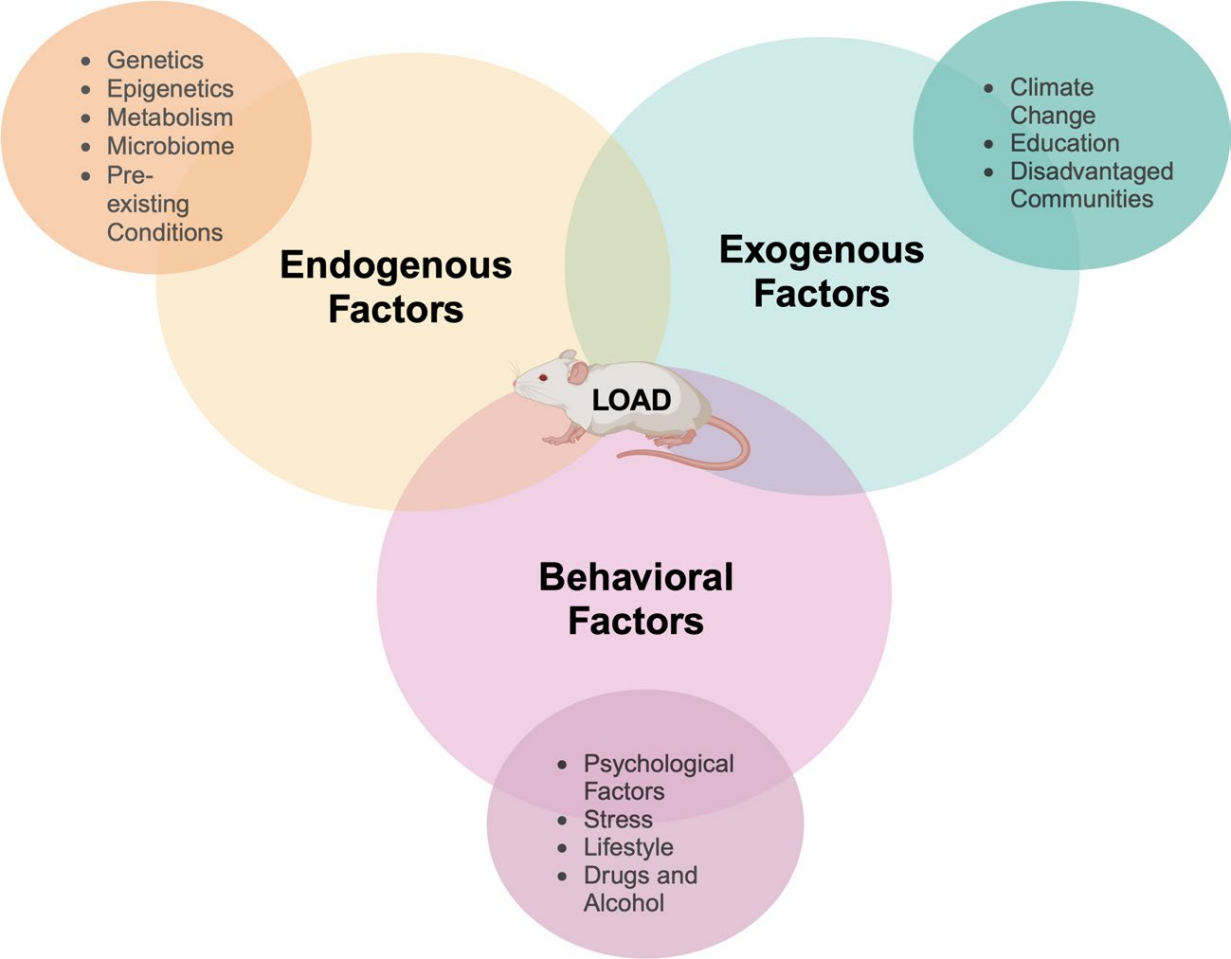
Infographic showing the relationship between the neural exposome factors and LOAD mouse models. Each factor alone can affect the LOAD mouse model but it is of greater importance to understand how all the factors together affect the progression and onset of ADRD in LOAD mice which can eventually be translated to humans

With new insights relating to ADRD being influenced by multiple factors, there is a need to develop mouse models for ADRD that account for the numerous mechanisms and pathways, as opposed to focusing on single aspects of ADRD, as observed in traditional ADRD models [157] (Fig. 5). Although traditional ADRD mouse models that account for Aβ and tau pathology have allowed for the experimental study of ADRD thus far, as observed in the aforementioned studies, it has become increasingly evident that these factors do not fully reflect the biology of ADRD. In many cases, mouse models that solely incorporate Aβ and tau pathology do not develop neurodegeneration and do not fully recapitulate the human neuropathology phenotype observed in LOAD, ultimately limiting extrapolation of studies in experimental mouse models to humans [157]. Therefore, attention has turned to the development of novel LOAD mouse strains that are more indicative of pathological changes observed in ADRD. LOAD is regarded to be the most common form of ADRD and is a complex pattern involving genetic and environmental exposures across one’s lifetime that can influence ADRD risk. APOE is one of few genes that have become an established LOAD genetic risk factor [158]. Specifically, the ε4-allele of APOE has been indicated to increase the risk of AD by about fourfold when inherited in one copy and over tenfold for two copies [158]. The Model Organism Development and Evaluation for Late-onset AD (MODEL-AD) Consortium is currently developing LOAD mouse models that can act as a platform strain for the addition of environmental and genetic risk factors to more closely mimic LOAD experimentally [159]. The LOAD2 mouse model includes humanized Aβ and two risk factors for ADRD, namely APOE ε4 and TREM2*R47H, that are incorporated into C57BL/6 J mice, ultimately producing a triple homozygous LOAD mouse model [159].

### The Neural Exposome and LOAD Mouse Models as an Approach to Modeling Disadvantaged Communities for the Study of ADRD

Through the findings of the aforementioned studies, the role of exposomic factors playing a role in ADRD cannot be undermined. Based on experiments that were originally performed using traditional ADRD mouse models to study the effect of the neural exposome factors on ADRD, these experiments can now utilize LOAD2 mice, to give novel insights into how environmental factors contribute to LOAD and can be used to simulate environments and conditions of disadvantaged communities. For instance, it has been suggested that individuals within a low SES community have distinct changes in their microbiome; more specifically, they have lower gut microbiome diversity [160]. There are also associations showing a relationship between low income and higher prevalence of diabetes [161] and how low wealth communities are more likely to be located near busy roadways, transit depots, industrial facilities, power plants, and oil and gas operations, leading to exposure to harmful pollutants [162]. These are all neural exposome factors that have been studied independently in traditional ADRD mouse models. As past studies have performed FMT from Alzheimer’s patients to rats [163], one suggestion is to conduct a study with the LOAD2 mouse model as a platform strain. FMT from a cohort of individuals from a disadvantaged community can be transplanted into a LOAD2 mouse model to simulate the microbiome of an individual from a disadvantaged community. These mice can then be crossed with an ob/ob mouse or NSy mouse to induce diabetes pathology, as past studies have induced diabetes pathology in ADRD mice through this method [164]. Then, experiments can be performed where this mouse model is exposed to PM to simulate an environment with air pollution that disadvantaged communities are more exposed to. As the LOAD2 model serves as a platform strain, multiple factors of the neural exposome can be tested simultaneously to model members of a disadvantaged community. However, it is also important to note some exposome factors are not as easily incorporated such as race and ethnicity which cannot be directly modeled in animal models. While certain genes such as Trem2 have consistent expression based on race, this is preliminary and not transferable immediately to animal models [165]. Thus, when developing models such as LOAD2, it is imperative to recognize how many factors including educational enrichment and diet can be incorporated into study designs to model disadvantaged communities but ethnicity or ethnic distribution is not as easily modeled in vivo. Nevertheless, in addition to animal models, the development of algorithms, and more importantly machine learning, could be a helpful tool to accelerate the prediction of ADRD onset and progression.

## Integrating Neural Exposome Animal Models with Modern Technology

### Machine Learning and Its Application to the Study of ADRD

Given that the LOAD mouse model can be explored for various neural exposome factors within a single study, a dataset with diverse variables can be compiled from it. With the dataset including variables such as climate change, microbiome, metabolism, and psychological stressors, we can utilize machine learning (ML) to make predictions about ADRD onset and progression. ML can be leveraged with several benefits: (1) pattern recognition—ML can identify patterns, trends, and correlations to understand the structure of complex datasets; (2) feature selection—ML can select the most essential features while dealing with diverse variables. Feature selection can not only simplify problems but also enhance efficiency; (3) discovering complex relationships—complex nonlinear relationships may exist among diverse variables, which can be challenging to handle using traditional statistical analysis. Utilizing ML models such as decision trees, support vector machines, and Gaussian process models allows interpreting these nonlinear relationships [166]. As hardware computing power becomes more robust, the application of ML can be employed in different biomedical areas, such as image recognition, voice recognition, and predictive analysis. Image recognition is based on the ML and combines a multilayer neural network learning algorithm to catalog a feature or an object from the digital image. This application can be used medically to determine whether there are signs of disease in patient X-rays [167]. Following this, audio analysis can address voice and speech with the ML model. In the biomedical field, a literature review identified that audio analysis can be used in diagnosing and monitoring voice-affecting disorders [168]. Using predictive analysis in future studies, we could use this form of ML to analyze patients’ electronic health records to predict disease risk and progression. A system review showed the EHR-based predictive models provided promising results, with 69% clinical improvement after model implementation [169]. In the realm of ADRD, ML emerges as a potential tool for constructing predictive models, surpassing the limitations of traditional statistical methods, which can only sufficiently summarize information derived from raw data. Diagnosing ADRD in its early stages is challenging, however, implemented ML models in the Open Access Series of Imaging Studies (OASIS) data demonstrate improved outcomes, achieving the highest validation average accuracy of 83% on the dataset [170]. We recognize the potential of employing ML as a strategy to mitigate ADRD. There are other studies that showcase the predictive capabilities of ML models when applied to each factor of the neural exposome. Through this, ML can allow for a comprehensive approach and understanding of the potential approaches related to the exogenous, endogenous, and behavioral factors within the neural exposome.

### Machine Learning Models for Exogenous, Endogenous, and Behavioral Factors

Elaborating on this discourse, ML can provide a new pathway to understanding and better implementing the use of the neural exposome into research regarding ADRD.

Past research using ADRD has focused on one factor of the three factors of the neural exposome. To elaborate, a study focused on the individual environmental and neighborhood factors and their role in ADRD because of the ten early symptoms of ADRD listed by The US Center for Disease Control and Prevention’s Healthy Brain Initiative and the Alzheimer’s Association, four of ten are related to spatial dysfunctions [171].

From classifying ML based on postal codes (zip codes), the study identified the significant impact of living environments on ADRD. Simultaneously, the study has also found a correlation between residing in rural areas and a higher risk of developing ADRD. Overall, the study provides crucial insights into the exogenous neural exposome factor and solidifies the knowledge base in the respective domain. A study, which focused on an endogenous factor of the neural exposome, employed ML to detect transcriptomic data obtained from the blood of ADRD patients and individuals without dementia (non-ADRD) [172]. During the analysis process, different ML models were trained using various hyperparameters. The support vector ML method enabled them to achieve a receiver operating characteristic (ROC) score of 93% and an accuracy of 89%. High scores were also obtained using neural network and logistic regression ML methods. These ML models indicate that oxidative stress induced by Aβ is a key feature for the high-accuracy prediction of ADRD. Thus, by implementing a ML model, we can elucidate the relationship between complicated endogenous factors and ADRD. As mentioned in the previous section, various factors influence ADRD, and it is important to note that lifestyle choices play an essential role in the risk assessment and reduction of ADRD. We must also employ behavioral factors and ML to investigate ADRD which are encompassed under the behavioral factor of the neural exposome. The use of ML has become an emerging area of interest for the study of lifestyle changes and its correlation to ADRD risk. One approach has focused on developing models and algorithms to collect and classify data related to lifestyle status, including physical activity and excessive diet, using electronic health records of ADRD patients [173]. This could be used to strengthen associations between lifestyle and ADRD risk and train ML models to better understand how lifestyle influences ADRD, and potentially identify causal effects [173]. As we construct the clinical models, we can harness the power of ML to maximize their effectiveness and build a comprehensive model targeting the three neural exposome factors.

### Clinical Models

LOAD mouse models would in part help facilitate the development of humans, as the developing LOAD mouse models incorporate humanized versions of Aβ and neurofibrillary tangle pathology, as well as the APOE ε4 risk factor [159]. However, mouse models can only be beneficial to a limited extent [174]. The use of mouse models can be used in conjunction with ML to take an unprecedented approach to studying ADRD, as it is related to the neural exposome, resulting in a novel interdisciplinary approach. For instance, research has suggested that rather than trying to directly humanize animal models for ADRD experimentally, ML can be used to humanize the data obtained from the study of experimental animal models [174]. The advancements in ML and its application to the study of ADRD can facilitate the development of human models of ADRD. By accounting for endogenous, exogenous, and behavioral factors, all core components of the neural exposome, into ML models, these models can ultimately be used to predict how the combination of these factors can influence ADRD risk, onset, and progression.

### From Animal Models to Human Models with Machine Learning: a Translational Approach

**Fig. 6:**
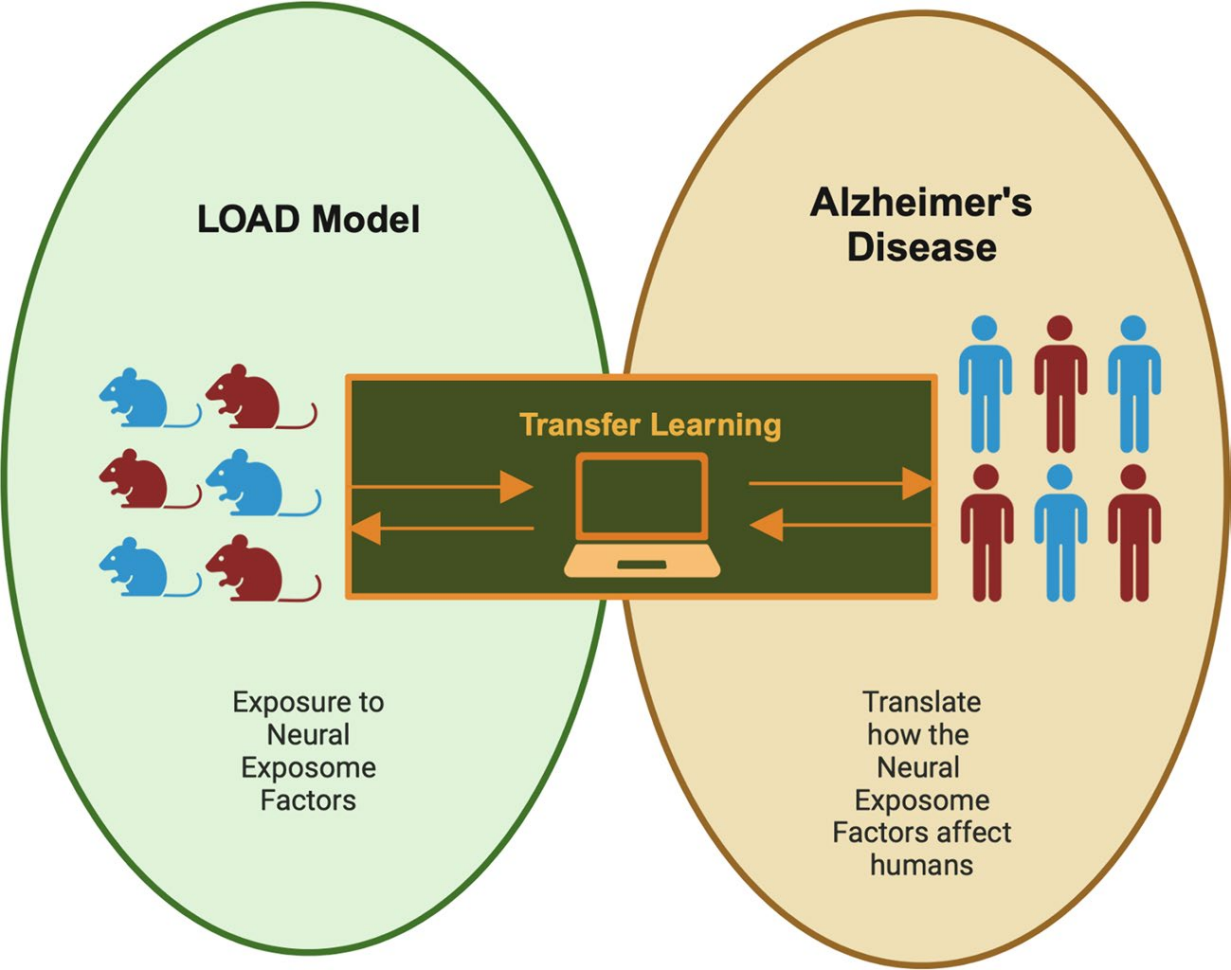
Infographic suggesting the relationship of how a LOAD mouse model can be translated to human Alzheimer’s disease through transfer learning. Through the exposure of LOAD mice to neural exposome factors we can better understand how these factors affect humans through translative machine learning

Previous research has focused on individual neural exposome factors. We propose to establish a comprehensive ML model targeting all three factors. As the traditional models focusing solely on Aβ and tau pathology may not fully reflect the complexity of ADRD, the LOAD mouse model can better mimic the pathological changes observed in the ADRD. From this mouse model, it provides us with a powerful tool to predict the interaction between the neural exposome and ADRD. In order to compare the results of animal models, the use of transfer learning (TL) to clinical models may be used (Fig. 6). TL is a ML technique that enhances data collection and learning by applying knowledge learned from one task to another related task [175]. TL offers more sensible, efficient, and practical answers by connecting earlier tasks even with a small data distribution (Fig. 6). TL offers numerous advantages in ML. Firstly, it enhances learning efficiency by leveraging knowledge from prior tasks, leading to quicker solutions. Secondly, TL improves model performance by incorporating insights from related tasks. Additionally, it diminishes the requirement for extensive datasets, making it particularly valuable in situations with limited availability of target training data. Lastly, TL is recognized for its ability to expedite output, contributing to faster and more efficient results in various applications. One study demonstrated the use of TL to map the bone marrow biology across different species with data derived from single-cell RNA sequencing [176]. After pre-processing and filtering, a total of 5504 cells were retained. The first step for the classifier was to build a multiclass logistic regression model to recognize different cell types, then the team converted the model to human equivalents. The next step was to restrict to those genes with a human orthologue and re-trained the original classifier with a limited set of human bone marrow cell gene expression signatures. Overall the accuracy reached 83% to identify individual human bone marrow cells, and the analysis showed how to map the biology across different species. An additional study utilized the TL through the application of computational pathology [177]. Due to the team’s restricted access to a human data set, they explored the use of the digital whole-slide images (WSIs) to segment glomeruli for human analysis. The outcome of the team showed promising results in the human glomerulus segmentation task. Overall, TL holds the potential to effectively utilize the transfer of models, serving as a promising approach to address and mitigate the challenges posed by ADRD. Unlike the traditional ML, we can improve the performance of the new task by leveraging the knowledge learned by the previous task, which can not only increase the productivity of the learning process but also produce output efficiency. Despite the valuable information provided by TL for physicians in disease diagnosis and treatment, safeguarding patient privacy data emerges as a critical concern in practical applications. Therefore, it is necessary to implement measures to strengthen the security and to mitigate the risk of privacy breaches. One example of how to protect patient confidentiality was implemented by the Private Aggregation of Teacher Ensembles (PATE) which ensures a better balance between the usability of data and the protection of patient privacy when constructing and utilizing medical models [178]. Overall, by introducing the optimization of ML models and TL, we can better understand the relationship between the neural exposome and ADRD, thereby enabling more effective mitigation of ADRD pathology.

## Conclusion

To conclude the discourse of the paper, the neural exposome is an important research and clinical tool that can facilitate the research of ADRD onset and progression. Additionally, we have highlighted the importance of ML in this process and how it can systematically and effectively improve and enhance research outcomes in animal models, and more specifically through the use of the LOAD model, and relate them to clinical models through the application of TL. The purpose of this paper was to expand broadly on the implications of the neural exposome, and its ability to improve and integrate multiple fields of study and prompt further communication within the scientific field.

## Data Availability

No datasets were generated or analysed during the current study.

## Abbreviations

AD: Alzheimer’s disease
ADRD: Alzheimer’s disease and related dementias
GABA: Gamma-aminobutyric acid
APOE: Apolipoprotein E
CSF: Cerebrospinal fluid
WHO: World Health Organization
ONETOX: Office of Neural Exposome and Toxicology Research
PM: Particulate matter
SES: Socioeconomic status
EOAD: Early-onset Alzheimer’s disease
APP: Amyloid precursor protein
PSEN1: Presenilin 1
PSEN2: Presenilin 2
LOAD: Late-onset Alzheimer’s disease
LPS: Lipopolysaccharide
BBB: Blood-brain barrier
Aβ: Amyloid β
COX: Cytochrome c oxidase
ABAD: Aβ-binding-alcohol-dehydrogenase
IGF: Insulin growth factor
T2D: Type-2 diabetes
MCI: Mild cognitive impairment
CUS: Chronic unpredictable stress
BZ: Benzodiazepines
WTCPM: World Trade Center Particulate Matter
DEE: Diesel engine exhaust
nPM: Nanoparticulate matter
FMT: Fecal matter transplantations
MODEL-AD: Model Organism Development and Evaluation for Late-onset AD
ML: Machine learning
OASIS: Open-access series of imaging studies
ROC: Receiver operating characteristic
TL: Transfer learning
WSIs: Whole slide images
PATE: Private Aggregation of Teacher Ensembles

## Declarations

### Disclaimer

We acknowledge that the contents of this study do not represent the views of the NCCIH, the ODS, the National Institutes of Health, the U.S. Department of Veterans Affairs, or the United States Government.
